# CKS2 and RMI2 are two prognostic biomarkers of lung adenocarcinoma

**DOI:** 10.7717/peerj.10126

**Published:** 2020-10-07

**Authors:** Dayong Xiao, Siyuan Dong, Shize Yang, Zhenghua Liu

**Affiliations:** 1 Department of Thoracic Surgery, The People’s Hospital of Wanning, Wanning, Hainan, China; 2 Department of Thoracic Surgery, The First Hospital of China Medical University, Shenyang, Liaoning, China

**Keywords:** Lung adenocarcinoma, CKS2, RMI2, Prognosis, Bioinformatics

## Abstract

**Background:**

Lung adenocarcinoma (ACA) is the most common subtype of non-small-cell lung cancer. About 70%–80% patients are diagnosed at an advanced stage; therefore, the survival rate is poor. It is urgent to discover accurate markers that can differentiate the late stages of lung ACA from the early stages. With the development of biochips, researchers are able to efficiently screen large amounts of biological analytes for multiple purposes.

**Methods:**

Our team downloaded GSE75037 and GSE32863 from the Gene Expression Omnibus (GEO) database. Next, we utilized GEO’s online tool, GEO2R, to analyze the differentially expressed genes (DEGs) between stage I and stage II–IV lung ACA. The using the Cytoscape software was used to analyze the DEGs and the protein-protein interaction (PPI) network was further constructed. The function of the DEGs were further analyzed by cBioPortal and Gene Expression Profiling Interactive Analysis (GEPIA) online tools. We validated these results in 72 pairs human samples.

**Results:**

We identified 109 co-DEGs, most of which were involved in either proliferation, S phase of mitotic cell cycle, regulation of exit from mitosis, DNA replication initiation, DNA replication, and chromosome segregation. Utilizing cBioPortal and University of California Santa Cruz databases, we further confirmed 35 hub genes. Two of these genes, encoding CDC28 protein kinase regulatory subunit 2 (CKS2) and RecQ-mediated genome instability 2 (RMI2), were upregulated in lung ACA compared with adjacent normal tissues. The Kaplan–Meier curves revealed upregulation of CKS2 and RMI2 are associated with worse survival. Using CMap analysis, we discovered 10 small molecular compounds that reversed the altered DEGs, the top five are phenoxybenzamine, adiphenine, resveratrol, and trifluoperazine. We also evaluated 72 pairs resected samples, results revealed that upregulation of CKS2 and RMI2 in lung ACA were associated with larger tumor size. Our results allow the deeper recognizing of the mechanisms of the progression of lung ACA, and may indicate potential therapeutic strategies for the therapy of lung ACA.

## Introduction

Lung malignant tumor is a significant global health issue. In 2018, 234,030 cases of lung and bronchus malignant tumor were reported the United States. Globally, the amounts of deaths from lung malignant tumor exceeds the number of deaths from prostate, colorectal, and breast cancer combined (Siegel, Miller & Jemal, 2019). Lung adenocarcinoma (ACA) is the most frequent histologic subtype and accounts about 40% of all lung cancers (Lawrence et al., 2013).

Because there are no obvious symptoms of early stage lung ACA, the five-year survival rate is about 18%. Optimal treatment of lung ACA requires accurate diagnosis and clinical staging before treatment begins (Shan et al., 2019). The anatomic basis for staging (tumors, lymph nodes, and metastases, TNM) includes the physical properties of the tumor and the presence of regional or systemic metastases. However, some shortcomings are associated with the current staging classification. The greatest limitation of conventional TNM staging is its inability to accurately distinguish high-risk patients, who are likely to develop metastasis, from low-risk patients, who will be complete cured after surgery. Hence, this limitation leads to some patients with metastases losing the opportunity of early intervention (Yang et al., 2018). One reason that high-risk and low-risk cases are difficult to distinguish is lung ACA is highly heterogeneous; hence, the morphology-based pathological stages and classifications are difficult to represent the heterogeneities (Yang et al., 2018). It is urgent to recognize the mechanisms underlying lung ACA progression, and search markers that can identify high-risk patients. The biologic basis for staging (molecular markers prognostic for survival, as well as indicators predictive for response to treatment) will be incorporated into staging systems in the future. Accurate staging of lung ACA patients before treatment will be helpful in predicting the prognosis for these patients.

With the rapid development of high-throughput bioinformatic technologies, differentially expressed genes (DEGs) can be widely screened and the potential functional pathways related to the genesis and prognosis of lung ACA can be identified. Our study identified 523 DEGs and 35 hub genes, and two of these genes (CKS2 and RMI2), are potential biomarkers for the prognosis of lung ACA. We further validated CKS2 and RMI2 using clinical biopsy samples and demonstrated that higher expression of these two genes correlates with larger tumor size and poor clinical outcomes. Using CMap analysis, we discovered 10 small molecular compounds that reversed the altered DEGs.

## Material and Methods

### Microarray data

The GSE75037 (Girard et al., 2016) and GSE32863 (Selamat et al., 2012) datasets were downloaded from GEO database, both of them were produced by Illumina HumanWG-6/Ref-8 v3.0, Expression BeadChip platform. The GSE75037 contains 83 cases of lung ACA and the GSE32863 contains 58 cases of lung ACA.

### Identification of DEGs

The GEO DataSets provide GEO2R, an online tool which can identify DEGs. A *p* -value of <0.05 and a logFC (fold change) of >0.5 were set as the cutoff values. Probe sets without exact gene symbols were excluded.

### KEGG and GO enrichment analyses of DEGs

The Database for Annotation, Visualization, and Integrated Discovery (DAVID, 6.8 version, http://david.ncifcrf.gov) and Kyoto Encyclopedia of Genes and Genomes (KEGG, https://www.genome.jp/kegg/) were adopted to extract biological information about the DEGs (Huang et al., 2007). Gene ontology (GO) is widely used in biological research (Le et al., 2019a; Le, Yapp & Yeh, 2019b).

### PPI network construction

We used Search Tool for the Retrieval of Interacting Genes (STRING, version 11.0, https://string-db.org/) to construct the protein-protein interaction (PPI) network from the DEGs (Szklarczyk et al., 2017). 0.4 was set as the minimum interaction score. The DEG molecular interaction network was constructed by Cytoscape (version 3.7.2, https://cytoscape.org/). The Molecular Complex Detection application was adopted to search the hub modules in the network. The inclusive parameters are set as follows: score >5, node score cutoff = 0.2, degree cutoff = 2, node density cutoff = 0.1, max depth = 100, and k-score = 2.

### Hub gene screen

Hub genes are those that have a degree >10 of intra-module connectivity. Hub genes were also screened utilizing Cytoscape software (version 3.7.2) (Dong et al., 2020; Li et al., 2017; Zhang et al., 2020). The visualization and functional process analysis were performed in Biological Networks Gene Oncology (BiNGO) (version 3.7.2).

### Functional analysis of hub genes in database

cBioPortal (http://www.cbioportal.org) was used to analyze the function of the hub genes (Cerami et al., 2012). The co-expression network of DEGs and mutations rates were also created by cBioportal.

University of California Santa Cruz (UCSC) Xena (https://xena.ucsc.edu/) is a functional genomics browser (Haeussler et al., 2019). The expression levels of the hub genes and the profiles of CKS2 and RMI2 in stage I–IV lung ACA were obtained from UCSC.

GEPIA is an online tool that provides the RNA sequencing expression data (Tang et al., 2017). The GEPIA database enables researchers to conduct multiple gene expression analyses. Relative expression level, Kaplan–Meier curves for overall survival and disease-free survival of stage I–IV lung ACA with CKS2 and RMI2 were obtained from GEPIA.

### Potential therapeutic agents

The Connectivity Map (https://www.broadinstitute.org/connectivity-map-cmap) is an online tool that analyses transcriptional data to explore the relationships between drugs and diseases (Lamb et al., 2006). We analyzed the DEGs by CMap to find potential therapeutic agents. The DEGS were input into the CMap website and the small molecular compound data were obtained. Screening criteria were set as follows: mean <−0.4 and *p* < 0.05.

### Validation in clinical samples

Lung ACA tissues (72 cases) and adjacent normal tissues were obtained from patients receiving surgery at the First Hospital of China Medical University between February 2013 and June 2014, the samples were confirmed by two pathologists. All resected tissues were stored in liquid nitrogen immediately until the RNA extraction was performed. The normal tissues were defined as three centimeters away from the margin of the tumor. There were 37 male and 35 female in our research, the age ranged from 38 to 75, with a median age of 60 years. The patients who had a history of cancer, chemo or radiotherapy were excluded. No significant correlation was found between RMI2 and CSK2 expression with age, gender, smoking history, lymph node metastasis, and distant metastasis.

CKS2 and RMI2 expression was detected using qPCR. We used TRIzol reagent (Invitrogen, Carlsbad, CA, USA) to isolate RNA. CKS2 (sense 5′- AGTTGTTGCCTGGGCTGGAC-3′and reverse, 5′-TCTCCTCCACTCCTCTTCAGACATC-3′) and RMI2 (sense, 5′-GAAAAACATCAAGATAAAGGACGCC-3′and reverse, 5′- GCAGAAACCCAACATTCAA AACC-3′) and GAPDH sense, 5′-CAATGACCCCTTCATTGA CC-3′and reverse, 5′-TGGAAGATGGTGATGGGATT-3′. The reaction was performed for 2 min at 50 °C, 10 min at 95 °C, 40 cycles at 95 °C for 15 s, and 60 °C for 30 s. 2–DDCT method was used to calculate the relative expression of RMI2 and CKS2 to GAPDH (Livak & Schmittgen, 2001). The Ethics Committee of the First Hospital of China Medical University approved this study (2017-75). Informed consent was obtained from all the included patients.

## Results

### Identification of DEGs in different stages of lung ACA

There were 50 cases of stage I lung ACA and 33 cases of stage II–IV lung ACA in the GSE75037 dataset, while the GSE32863 dataset contained 34 cases of stage I lung ACA and 24 cases of stage II–IV lung ACA.

**Figure 1:**
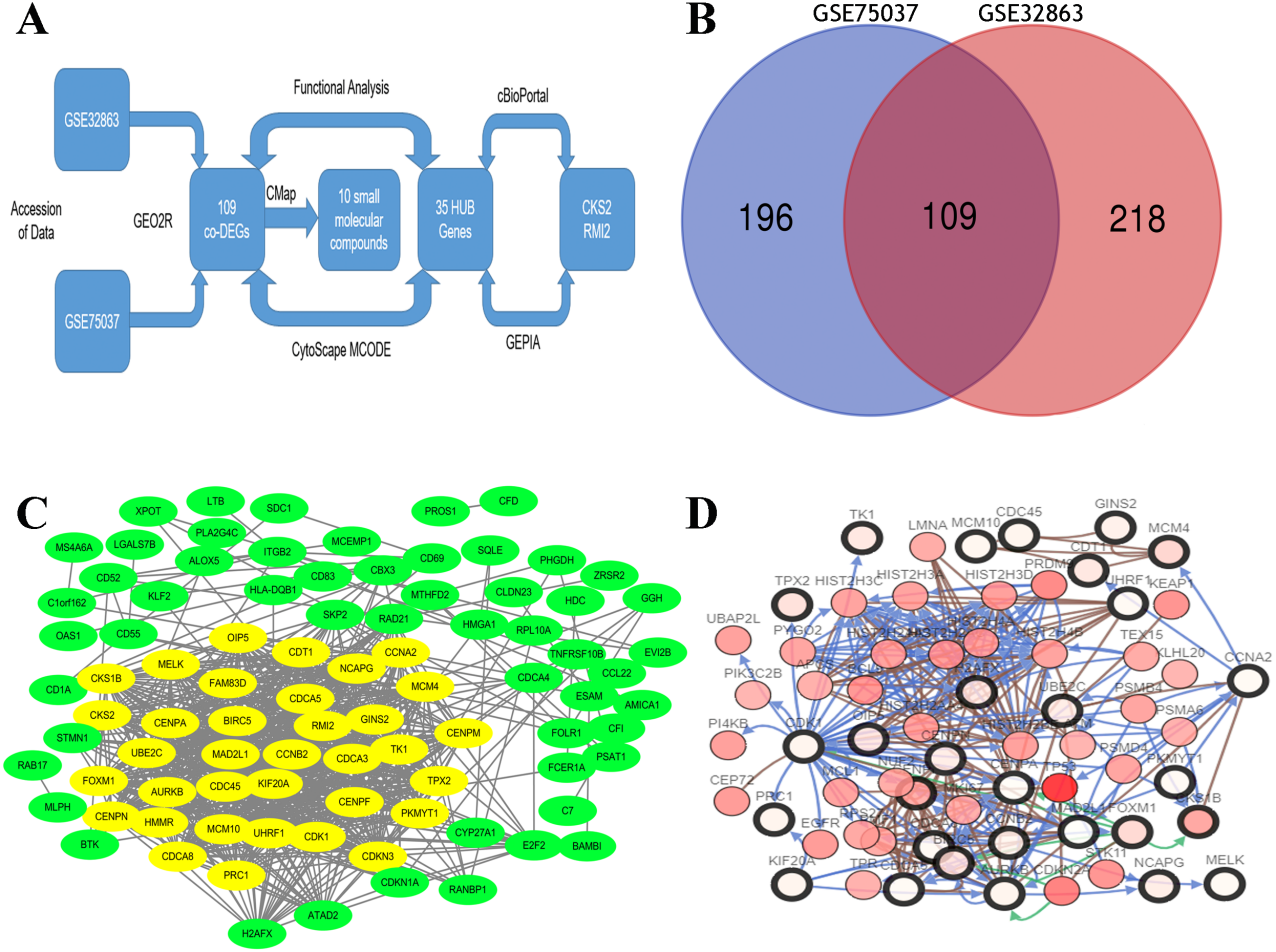
Flow-chart, Venn diagram, and interaction of the hub genes of this research. (A) Flow-chart of this research. (B) Two expression biochips, GSE328863 and GSE75037, were probed. 109 co-DEGs were identified. (C) Interaction network showing 35 hub genes. (D) Thirty-five hub genes and their co-expressed genes were built using cBioPortal.

By standardizing the data, there were 523 DEGs were found between stage I and stage II–IV lung ACA (305 in GSE75037 and 327 in GSE32863). A Venn diagram was constructed, which showed there were 109 co-DEGs between these two datasets (Fig. 1A).

### PPI network construction

A DEG PPI network (Fig. 1B) was created and the most significant genes (Fig. 1B, yellow) were identified by Cytoscape (Fig. 1B). Each DEG’s degree was calculated using CytoScape software. The degree indicates the number of connected nodes with each individual node, so a higher degree indicates a characteristic hub. The hub genes was defined as degree higher than 10 and thirty-five genes were identified. Similarly, the more central (closeness centrality) a cycle is, the closer it is to other cycles; so, high closeness centrality represents the tendency of a cycle to be a hub (Fig. 1B, yellow). The top 10 hub gene symbols, full names, functions, and degrees are listed in Table 1.

**Table 1:** The summary of top 10 hub genes.

NO	Gene symbol	Full name	Function	Degree
1	CDK1	Cyclin Dependent Kinase 1	Plays a key role in the control of the eukaryotic cell cycle by modulating the centrosome cycle as well as mitotic onset.	45
2	CDC45	Cell Division Cycle 45	An essential protein required to the initiation of DNA replication.	40
3	CENPF	Centromere Protein F	CENPF is a component of the nuclear matrix during the G2 phase of interphase.	37
4	TK1	Thymidine Kinase 1	A cytosolic enzyme that catalyzes the addition of a gamma-phosphate group to thymidine.	36
5	PRC1	Protein Regulator Of Cytokinesis 1	Key regulator of cytokinesis that cross-links antiparrallel microtubules.	34
6	CKS2	CDC28 Protein Kinase Regulatory Subunit 2	Binds to the catalytic subunit of the cyclin dependent kinases and is essential for their biological function.	32
7	CDCA3	Cell Division Cycle Associated 3	Acts by participating in E3 ligase complexes that mediate the ubiquitination and degradation of WEE1 kinase at G2/M phase.	30
8	ATAD2	ATPase Family AAA Domain Containing 2	A transcriptional coactivator of the nuclear receptor ESR1 required to induce the expression of a subset of estradiol target genes.	23
9	RMI2	RecQ Mediated Genome Instability 2	A complex that plays an important role in the processing of homologous recombination intermediates.	23
10	STMN1	Stathmin 1	Involved in the regulation of the microtubule (MT) filament system by destabilizing microtubules.	11

The network of the 35 hub genes and the co-expressed genes were built using cBioPortal (Fig. 1C). The red and black circled nodes are more important nodes, and we found that TP53 was present in the network.

### Functional analyses of the DEGs

**Figure 2:**
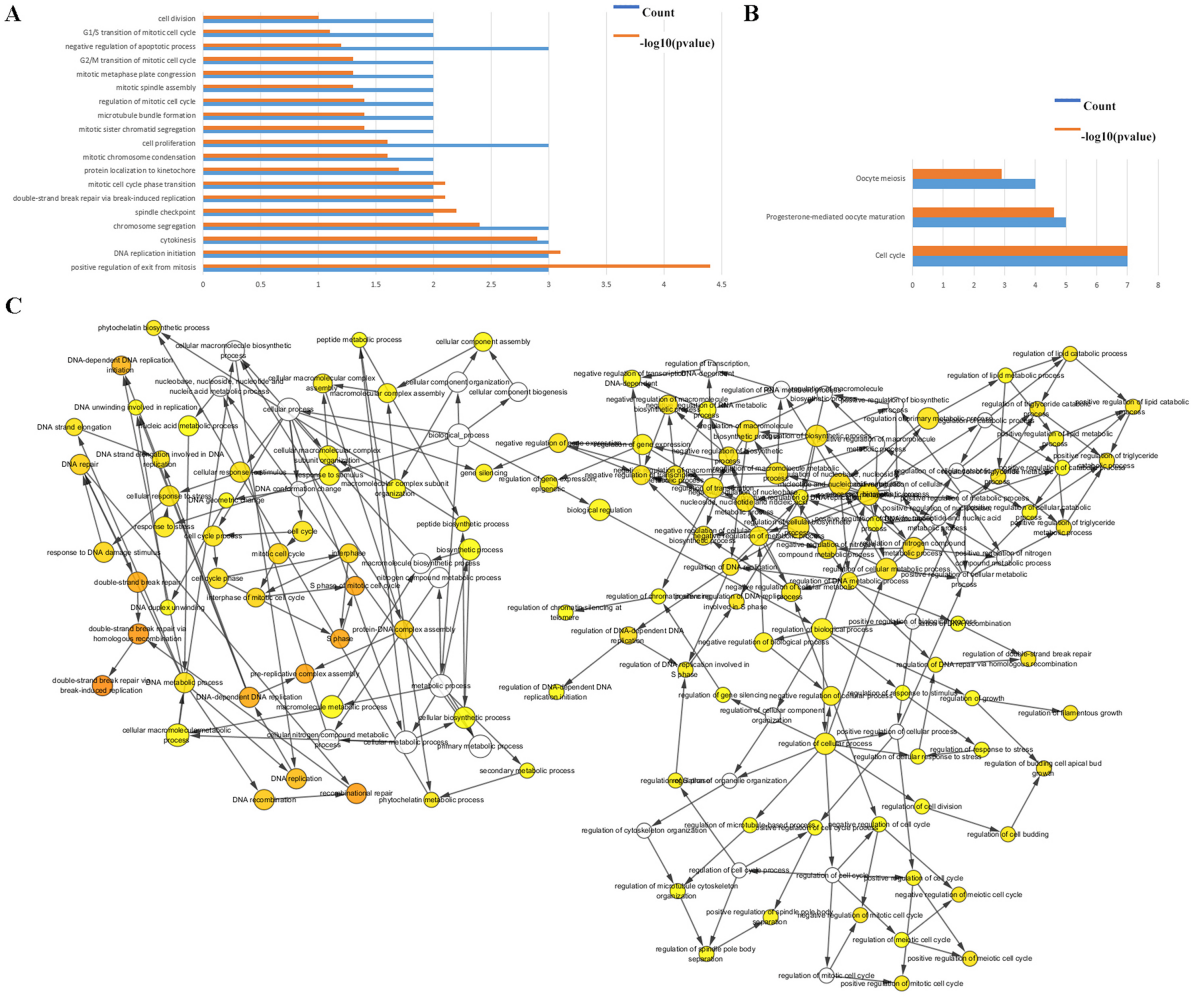
Functional analysis of the hub genes. (A & B) The functions of the 35 hub genes were analyzed by DAVID, and most of the genes were found to be implicated in cell apoptosis and cycle regulation. (C) The plugin of Cytoscape, BiNGO, was used to analyze the biological processes of the hub genes which are mainly involved in regulation of growth and cell cycle.

The biological classifications, functions, and pathway enrichment of the DEGs were researched using the DAVID online tool. The biological processes (BP) of the DEGs was analyzed by GO, and they were found to be mainly involved in the regulation of positive regulation of exit from mitosis, DNA replication initiation, proliferation, S phase of mitotic cell cycle, mitotic cell cycle transition, and chromosome segregation (Fig. 2A). By analyzing the cell components, the locations of DEGs were the cell surface, the interstitial matrix, the cytoplasm, and cell-substrate adherent junctions. DEGs were generally represented in the cell cycle, oocyte meiosis, and in progesterone-mediated oocyte maturation (Fig. 2B).

The BP were also analyzed by Cytoscape, the results also indicated the BP are involved in the cell cycle, S phase of mitotic cell cycle, regulation of cell budding and in DNA-dependent DNA replication initiation, these are all important cellular proliferative functions (Fig. 2C).

### Clinical significance of CKS2 and RMI2

**Figure 3:**
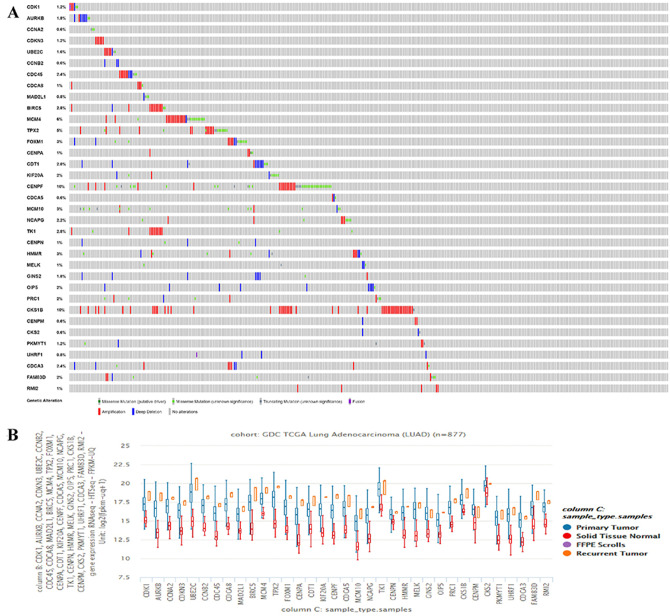
The hub genes’ mutation rates and expression levels in lung ACA. (A) The cBioPortal database shows that the 35 hub genes are known to be mutated in lung ACA. (B) The UCSC database shows that all hub genes are more highly expressed in both primary tumors and relapse tumors compared to normal lung tissues.

The hub genes’ genetic mutation rates were analyzed by cBioPortal, the results revealed that the CKS1B has the highest genetic mutation rates, the CKS2 and RMI2 are 0.6% and 1%, respectively (Fig. 3A). The expression level of the 35 hub genes in primary tumor, recurrent, and normal lung tissues were also analyzed. Results revealed both the CKS2 and RMI2 are higher expressed in tumor than normal lung tissues (Fig. 3B).

There are 109 co-DEGs between the GSE75037 and GSE32863 datasets, and CKS2 and RMI2 had the relative high degrees at 32 and 23 (Table 1), manifesting that these two genes act a pivotal part in the genesis and progression of tumor. The top 10 hub genes are listed in Table 1. At present, there are no studies that investigate the relationship between CKS2 and RMI2 and the prognosis of lung ACA.

**Figure 4:**
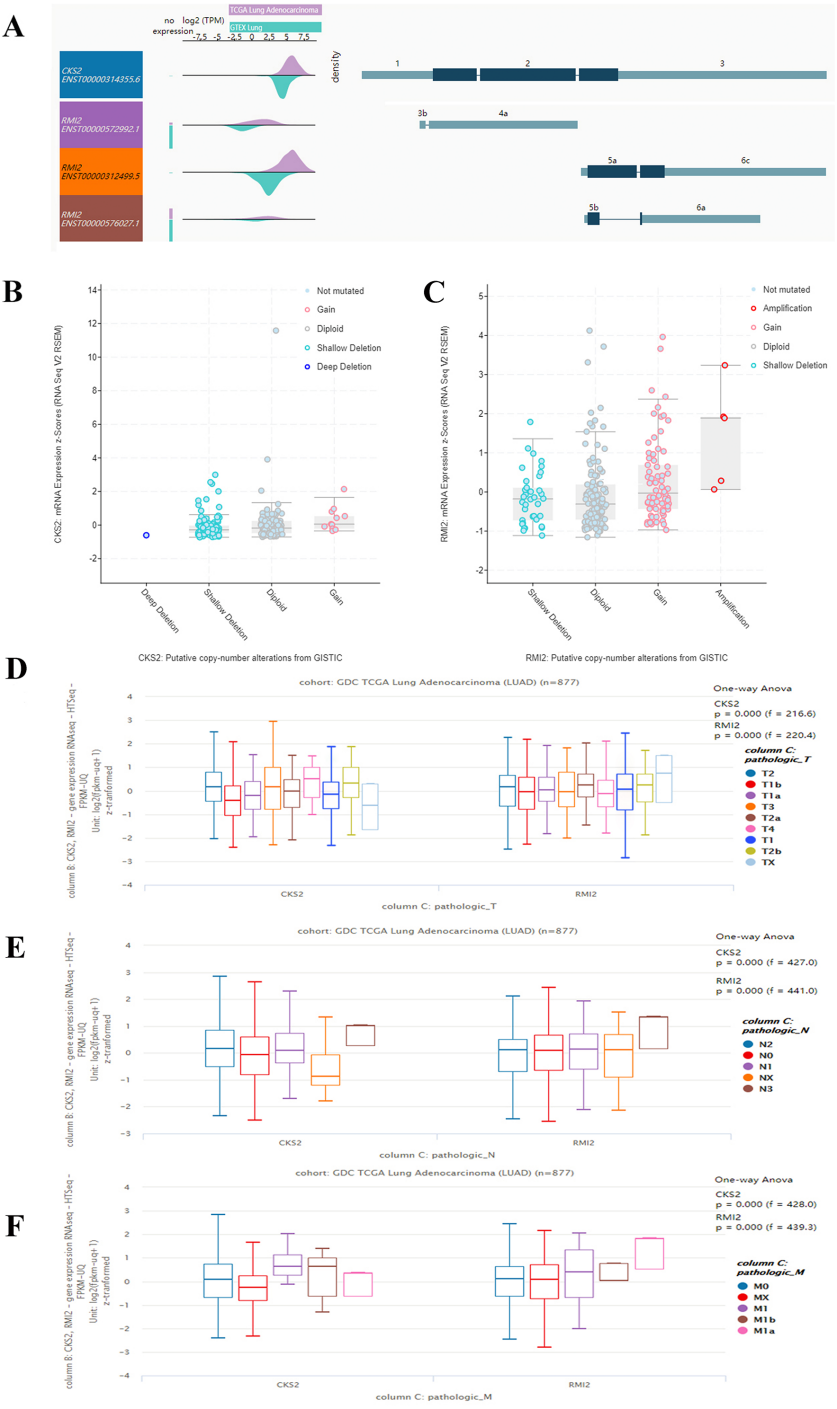
Expression of CKS2 and RMI2 is elevated in lung ACA. (A) The UCSC database showed that the transcripts of both CKS2 and RMI2 are over-expressed and mutated in lung ACA. (B and C) The mutation status of CKS2 in lung ACA is mainly shallow deletion, and the RMI2 is mainly amplification. With the upgrade of T (D), N (E), and M (F) staging, the expression levels of CKS2 and RMI2 also increased.

By querying UCSC data, the expression levels of CKS2 and RMI2 in lung ACA were found to be higher than those in lung tissues (Fig. 4A). And the mutation status of CKS2 in lung ACA is mainly shallow deletion (Fig. 4B), and that of RMI2 is mainly amplification (Fig. 4C). Our team further probed the expression levels of CKS2 and RMI2 in different TNM stages of lung ACA. With the upgrade of T, N, and M stages, expression levels of CKS2 and RMI2 also increased (Figs. 4D, 4E and 4F).

**Figure 5:**
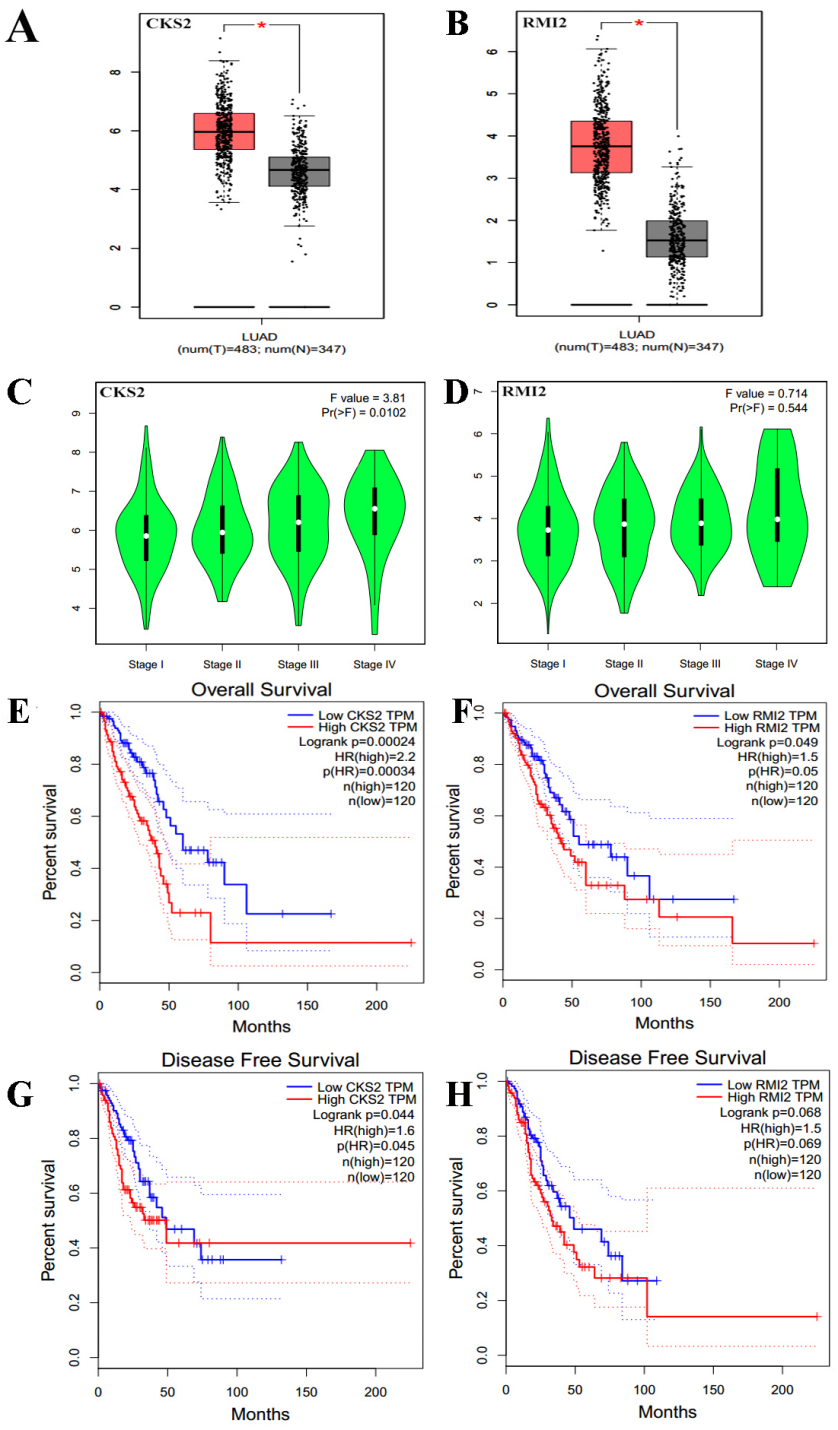
Clinical significance of CKS2 and RMI2 in lung ACA. Both CKS2 (A) and RMI2 (B) in lung ACA are significantly higher than in normal lung tissues. Along with the pathological staging of lung ACA, the expression levels of CKS2 (C) and RMI2 (D) also increased. Lung ACA patients with higher levels of CKS2 (E) and RMI2 (F) expression had worse overall survival rates. Patients with higher levels of CKS2 (G) expression had worse disease-free survival rates; no difference was observed in the RMI2 (H) group.

The expression levels of CKS2 and RMI2 in lung ACA were investigated using the GEPIA database. We found that the expression of both CKS2 and RMI2 in lung ACA are higher than in normal lung tissues (Figs. 5A and 5B). Staging of lung ACA is one of the important determinants of tumor prognosis. Along with the pathological staging of lung ACA, the expression levels of CKS2 and RMI2 increased (Figs. 5C and 5D).

The relationships between CKS2 and RMI2 and disease-free survival and overall survival were analyzed utilizing Kaplan–Meier curves. The results showed that lung ACA patients with relative higher levels of CKS2 and RMI2 expression had worse overall survival rates (Figs. 5E and 5F), and patients with higher expression levels of CKS2 even had worse disease-free survival rates (Fig. 5G).

### Potential therapeutic agents

Using CMap analysis, we found 10 small molecular compounds that could reverse the altered DEGs, the top 5 are phenoxybenzamine, adiphenine, resveratrol, trifluoperazine, (Table 2). These compounds have potential for lung ACA treatment. However, the mechanism of these drugs to reverse the altered DEGs are still unclear, further researches are needed.

**Table 2:** Small molecular compounds to reverse the altered DEGs by CMap analysis.

Rank	CMap name	Mean	n	Enrichment	p	Specificity	Percent non-null
1	phenoxybenzamine	−0.775	4	−0.955	0	0.0091	100
2	adiphenine	0.746	5	0.946	0	0	100
3	resveratrol	−0.756	9	−0.887	0	0	100
4	trifluoperazine	−0.513	16	−0.552	0	0.0577	75
5	prochlorperazine	−0.543	16	−0.549	0	0.0377	93
6	trichostatin A	−0.412	182	−0.33	0	0.4654	73
7	estradiol	0.218	37	0.375	0.00004	0.044	51
8	acepromazine	−0.744	4	−0.916	0.0001	0	100
9	pentoxifylline	0.632	5	0.852	0.00018	0	100
10	medrysone	−0.714	6	−0.784	0.00018	0.0108	100

### Validation in human samples

**Figure 6:**
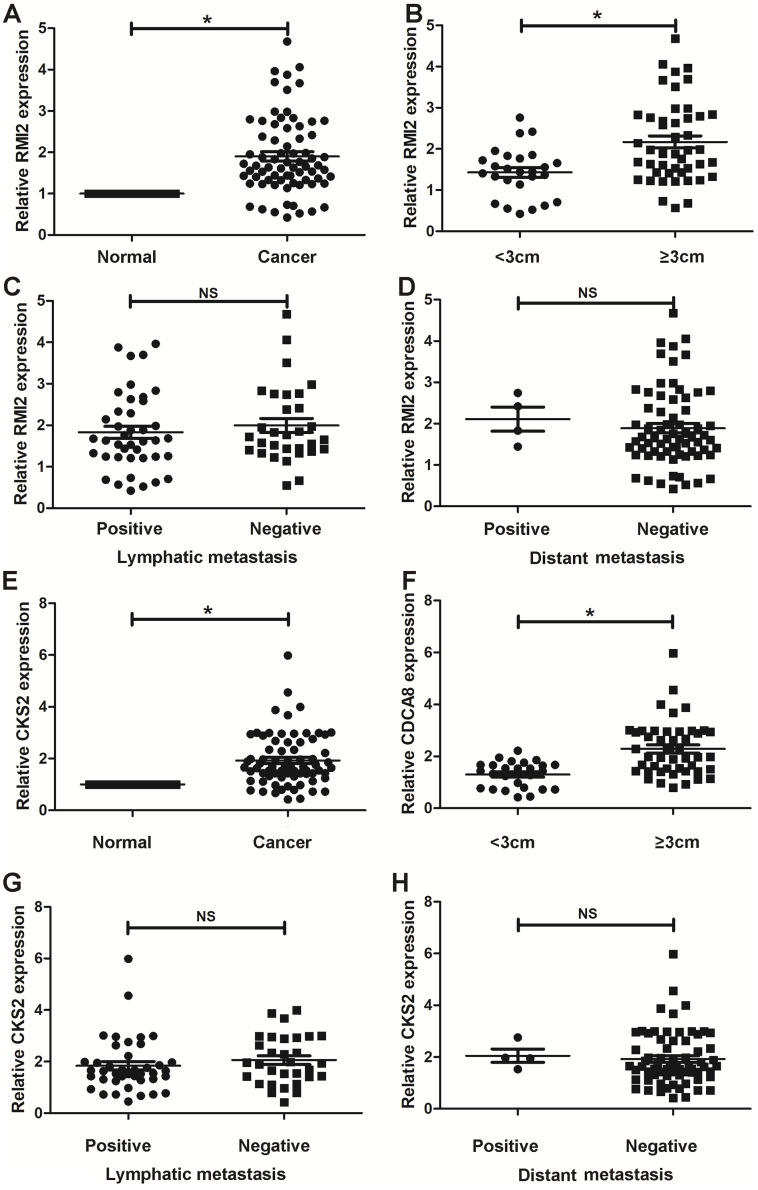
Validation in human samples. CKS2 and RMI2 were validated by 72 pairs of clinical biopsy specimens. qPCR was used to determine the correlation between CKS2 (A) and RMI2 (E) expression and tumor presence, tumor size (B and F), lymph metastasis (C and G), and distant metastasis (D and H). ^∗^*p* < 0.05.

Thus far, we have determined that CKS2 and RMI2 are two potential diagnostic markers for lung ACA. This was determined using a number of applied bioinformatics strategies. We chose to further validate these results using clinical samples taken from patients with lung ACA as well as matching adjacent normal tissue. The results from the qPCR aligned with our bioinformatic results, suggesting that lung ACA samples have higher levels of CKS2 and RMI2 than normal tissue (*P*<0.05, Figs. 6A and 6E). While there was no significant difference due to lymphatic metastasis and distant metastasis (Figs. 6C–6D and 6G–6H), there were differences related to tumor size (*P* < 0.05, Figs. 6B and 6F). These results suggest CKS2 and RMI2 are two valuable markers which can be used to assist in the diagnosis of lung ACA.

## Discussion

Lung ACA is the most common subtype of non-small cell lung cancer which is featured by distinct molecular characteristics. Lung ACA is often advanced by the time it is diagnosed (Lawrence et al., 2013). Moreover, lung ACA is highly heterogeneous at multiple levels. Traditional TNM staging alone is challenging to predict how the disease will behave (Yang et al., 2018). Early lung cancer has a satisfactory prognosis; however, in some patients lung ACA usually returns within three years in distant locations after receiving surgery, even though the tumors are not big. In order to reduce the risk of recurrence, in addition to surgery, closer follow-up and adjuvant therapy are needed for these high-risk patients. It is therefore important to identify aggressive tumors before treatment and after surgery.

In an era of increasingly complex treatment options (sometimes for molecular events that lead to cancer) and the need to obtain maximum information from minimally invasive samples, assistive technologies have been developed to improve the specificity of diagnosis. (Yang et al., 2018). According to cell-specific antigen expression and genetic information changes, the diagnosis and prognosis of the disease can be predicted. (Oellerich et al., 2017).

In our research, two mRNA microarray datasets were downloaded and analyzed to acquire co-DEGs between stage I lung ACA and stage II–IV lung ACA. KEGG and GO enrichment analyses were used to probe the functions of these DEGs. The co-DEGs were found to be mainly take part in the positive regulation of exit from mitosis, DNA replication initiation, proliferation, S phase of mitotic cell cycle, mitotic cell cycle transition, cellular component assembly, and chromosome segregation. These pathways are closely associated with tumor genesis and progression (Blackford & Stucki, 2020; Venuto & Merla, 2019). Hence, there finding are consistent with previous studies and theories.

In this research, the degree shows the number of nodes connected with the individual node, 35 genes with degrees of at least 10 were deemed as hub genes. Therefore, a higher degree indicates a characteristic hub and also a critical role in the genesis and progression of lung ACA.

To confirm the critical roles of 35 hub genes in the genesis and progression of lung ACA, the expression levels of these genes were further investigated using the UCSC online tool. The results revealed that all of these genes were higher expressed in both primary and recurrent tumors than normal lung tissues. The relative expression levels of the DEGs in primary tumors were lower than in recurrent tumors which suggests that these genes can be used as indicators to monitor tumor recurrence. The results indicate that these genes play a critical role in the occurrence and development of lung ACA. These genes may therefore be regarded as early biomarkers to monitor tumor recurrence.

The relationship between CKS2 and RMI2 and lung cancer has not yet been reported. Therefore, we further studied the relationship between these two genes and lung ACA in order to find novel tumor markers related to the prognosis of lung ACA.

The CKS2 protein binds to the catalytic subunit of cyclin dependent kinases and is dispensable for the function. In HeLa cells, different patterns of the mRNA is discovered throughout the cell cycle, indicating an essential part for the encoded protein (Nebreda & Ferby, 2000). The high expression of CKS2 is related with the progression of bladder cancer and hepatocellular carcinoma (Kawakami et al., 2006; Shen et al., 2010). In our research, CKS2 interacts with maternal embryonic leucine zipper kinase and Forkhead Box M1 (FOXM1), which are closely related with malignant tumors, indicating that CKS2 plays an important role in lung ACA. Our study also revealed the expression level of CKS2 in lung ACA is higher than in healthy lung tissue. Moreover, as the T stage of lung ACA escalates, the expression of CKS2 also increases. The same results were also seen in the association between N and M staging and CKS2 expression levels. The survival rate and disease-free survival rate of lung ACA with higher expression of CKS2 are significantly lower than that of lung ACA with lower expression of CKS2 (Figs. 5E and 5G).

RMI2 is a eukaryotic family of OB3, oligo-nucleotide-binding proteins. It is an dispensable component of the RMI complex and plays a vital part in the producing of homologous recombination intermediates in order to control DNA-crossover-formation in cells (Wang et al., 2010). The mutation of RMI2 is associated with Bloom syndrome, a recessive human genetic disease with features of and predisposition to cancer (Xu et al., 2008). In our research RMI2 interacts with cell division cycle-associated protein 3 (CDCA3) and cyclin B2 (CCNB2), which are also related with malignant tumors. CDCA3 and CCNB2 function as regulatory proteins and interact with other proteins at some vital phases in the cell cycle that play a role in tumorigenesis. At present, no researches reported the relationship between RMI2 and cancer. Our results show that the expression level of RMI2 in lung ACA is higher than that in healthy lung tissue. Moreover, as the T, N and M stage of lung ACA escalates, the expression of RMI2 also increases. The survival rates of lung ACA with higher expressions of RMI2 are significantly lower than that of lung ACA with lower expression of RMI2, but there is no difference in the fields of disease-free survival between these two groups. We inferred that the higher RMI2 group is not sensitive to further treatment once recurrence occurs, although the underlying mechanism is not clear. We chose to further validate these results using clinical samples taken from patients with lung ACA and compared them to matching adjacent normal tissue, and the results confirmed the bioinformatics conclusions.

The carcinoembryonic antigen (CEA) had been used as biomarker of lung adenocarcinoma for many years. Tevfk’s research reveals carcinoembryonic antigen (CEA) can get high concentrations in the pleural fluid and blood (Akcam et al., 2017). However, there is no specific biomarker for lung adenocarcinoma currently. Different with previous study, with development of biochip, more biomarkers can be found. We hope to discover a specific lung adenocarcinoma biomarker to predict the prognosis.

Next, we conducted a clinical translational study based on the DEG results. In this research, 10 small molecular compounds were found to reverse the altered DEGs, and therefore could potentially be used for the treatment of lung ACA. These compounds included phenoxybenzamine, adiphenine, resveratrol, trifluoperazine, and prochlorperazine. The antitumor effects of resveratrol have already been reported for several malignant diseases, including lung cancer (Rauf et al., 2018). Rasheduzzaman’s research revealed that resveratrol sensitizes NSCLC cells to TNF-related apoptosis-inducing ligand via p53-independent signaling and the inhibition of Akt/NF-κB pathway (Rasheduzzaman, Jeong & Park, 2018). Another study reported that resveratrol generates protective autophagy in NSCLC via suppression of Akt/mTOR signaling and activation of p38-MAPK pathway (Wang et al., 2018). The above articles support the results of our research. The inhibitory effects of other drugs on lung cancer have not yet been reported and require further research.

## Conclusions

The goal of this research was to screen novel biomarkers of lung ACA, which may become valuable prognostic and therapeutic targets. We also evaluated two unique microarrays from GEO and identified 35 highly connected DEGs that were discovered to be upregulated in the stage II–IV lung ACA. Through functional analysis, we focused on CSK2 and RMI2 as previously under-represented markers of lung ACA. Further study is desired to clarify the underlying molecular mechanisms behind the alteration in expression of CKS2 and RMI2 in lung ACA and their biological functions.

##  Supplemental Information

10.7717/peerj.10126/supp-1 Supplemental Information 1 STRING

10.7717/peerj.10126/supp-2 Supplemental Information 2 STRING data

10.7717/peerj.10126/supp-3 Supplemental Information 3 GEO data

10.7717/peerj.10126/supp-4 Supplemental Information 4 PCR results

10.7717/peerj.10126/supp-5 Supplemental Information 5 Raw data of GO and KEGG

10.7717/peerj.10126/supp-6 Supplemental Information 6 CM’a’p

10.7717/peerj.10126/supp-7 Supplemental Information 7 Hub genes’ function

## Additional Information and Declarations

### Data Availability

The following information was supplied regarding data availability: The raw measurements are available in the Supplementary Files.
