# Supplementary material for: CKS2 and RMI2 are two prognostic biomarkers of lung adenocarcinoma
Source: PeerJ. 2020 Oct 7;8:e10126. doi: 10.7717/peerj.10126 (PMC7547618; doi:10.7717/peerj.10126)
Supplement: Supplemental Information 7 [file peerj-08-10126-s007.docx]

| NO | Gene symbol | Full name | Function | Degree |
| --- | --- | --- | --- | --- |
| 1 | CKS1B | CDC28 Protein Kinase Regulatory Subunit 1B | Binds to the catalytic subunit of the cyclin dependent kinases and is essential for their biological function. | 33 |
| 2 | MELK | Maternal Embryonic Leucine Zipper Kinase | Calcium ion binding and protein kinase activity | 35 |
| 3 | OIP5 | Opa Interacting Protein 5 | Essential for recruitment of CENP-A through the mediator Holliday junction recognition protein. | 34 |
| 4 | CDT1 | Chromatin Licensing And DNA Replication Factor 1 | Involved in the formation of the pre-replication complex that is necessary for DNA replication. | 37 |
| 5 | NCAPG | Non-SMC Condensin I Complex Subunit G | Responsible for the condensation and stabilization of chromosomes during mitosis and meiosis. | 36 |
| 6 | CCNA2 | Cyclin A2 | Regulators of the cell cycle. | 42 |
| 7 | MCM4 | Minichromosome Maintenance Complex Component 4 | Initiation of eukaryotic genome replication. | 38 |
| 8 | CENPM | Centromere Protein M | Binds spindle microtubules to regulate chromosome segregation | 33 |
| 9 | TPX2 | TPX2 Microtubule Nucleation Factor | Required for normal assembly of microtubules during apoptosis. | 38 |
| 10 | PKMYT1 | Protein Kinase, Membrane Associated Tyrosine/Threonine 1 | Negatively regulates the G2/M transition of the cell cycle. | 32 |
| 11 | CDKN3 | Cyclin Dependent Kinase Inhibitor 3 | Cyclin-dependent kinase inhibitor. | 42 |
| 12 | GINS2 | GINS Complex Subunit 2 | Essential for the initiation of DNA replication | 34 |
| 13 | CDCA5 | Cell Division Cycle Associated 5 | Chromatin binding. | 37 |
| 14 | CCNB2 | Cyclin B2 | Member of the cyclin family, specifically the B-type cyclins. | 41 |
| 15 | KIF20A | Kinesin Family Member 20A | Protein kinase binding and ATPase activity. | 37 |
| 16 | CDCA8 | Cell Division Cycle Associated 8 | Regulator of mitosis and cell division. | 40 |
| 17 | CENPA | Centromere Protein A | Specify the mitotic behavior of chromosomes. | 37 |
| 18 | FAM83D | Family With Sequence Similarity 83 Member D | Regulates cell proliferation, growth, migration and epithelial to mesenchymal transition. | 25 |
| 19 | BRIC5 | Baculoviral IAP Repeat Containing 5 | Negative regulatory proteins that prevent apoptotic cell death. | 40 |
| 20 | MAD2L1 | Mitotic Arrest Deficient 2 Like 1 | Component of the mitotic spindle assembly checkpoint | 40 |
| 21 | UBE2C | Ubiquitin Conjugating Enzyme E2 C | Targeting abnormal or short-lived proteins for degradation. | 41 |
| 22 | AURKB | Aurora Kinase B | Regulation of alignment and segregation of chromosomes during mitosis and meiosis | 42 |
| 23 | MCM10 | Minichromosome Maintenance 10 Replication Initiation Factor | Initiation of eukaryotic genome replication. | 36 |
| 24 | FOXM1 | Forkhead Box M1 | Transcriptional activator involved in cell proliferation. | 38 |
| 25 | HMMR | Hyaluronan Mediated Motility Receptor | Involved in cell motility. | 35 |
